# S100A9/CD163 expression profiles in classical monocytes as biomarkers to discriminate idiopathic pulmonary fibrosis from idiopathic nonspecific interstitial pneumonia

**DOI:** 10.1038/s41598-021-91407-9

**Published:** 2021-06-09

**Authors:** Masahiro Yamashita, Yuh Utsumi, Hiromi Nagashima, Hiroo Nitanai, Kohei Yamauchi

**Affiliations:** 1 Department of Pulmonary Medicine, Allergy and Immunological Diseases, Iwate Medical University School of Medicine, 1-1-1 Idaidouri, Yahabacho, Shiwa, 028-3694 Japan; 2 Internal Medicine, Ishidoriya Medical Center, Hanamaki, Japan; 3 Internal Medicine, Takisawa Chuo Hospital, Takisawa, Japan

**Keywords:** Diagnostic markers, Respiratory tract diseases

## Abstract

Circulating monocytes have pathogenic relevance in idiopathic pulmonary fibrosis (IPF). Here, we determined whether the cell surface levels of two markers, pro-inflammatory-related S100A9 and anti-inflammatory-related CD163, expressed on CD14^strong^CD16^−^ classical monocytes by flow cytometry could discriminate IPF from idiopathic nonspecific interstitial pneumonia (iNSIP). Twenty-five patients with IPF, 25 with iNSIP, and 20 healthy volunteers were prospectively enrolled in this study. The S100A9^+^CD163^−^ cell percentages in classical monocytes showed a pronounced decrease on monocytes in iNSIP compared to that in IPF. In contrast, the percentages of S100A9^−^CD163^+^ cells were significantly higher in iNSIP patients than in IPF patients and healthy volunteers. In IPF patients, there was a trend toward a correlation between the percentage of S100A9^+^CD163^−^ monocytes and the surfactant protein-D (SP-D) serum levels (*r* = 0.4158, [95% confidence interval (CI) − 0.02042–0.7191], *p* = 0.051). The individual percentages of S100A9^+^CD163^−^ and S100A9^−^CD163^+^ cells were also independently associated with IPF through multivariate regression analysis. The unadjusted area under the receiver operating characteristic curve (ROC-AUC) to discriminate IPF from iNSIP was (ROC-AUC 0.802, 95% CI [0.687–0.928]), suggesting that these are better biomarkers than serum SP-D (*p* < 0.05). This preliminary study reports the first comparative characterization of monocyte phenotypes between IPF and iNSIP.

## Introduction

Idiopathic pulmonary fibrosis (IPF) is a chronic and progressive fibrogenic disease of unknown etiology, and the prognosis of IPF remains unsatisfactory^1^. Aberrant healing of lung injury is believed to be a cause of fibrogenesis, and reactive oxygen species (ROS) are involved in tissue damage^2,3^. Idiopathic nonspecific interstitial pneumonia (iNSIP) is also characterized by chronic and treatment-reversible fibrotic lung conditions, but its prognosis is excellent. The mechanisms underlying the prognostic differences between IPF and iNSIP as well as their pathogenesis, remain to be elucidated. Early intervention using anti-fibrotic agents can improve the prognosis of IPF^4^. However, the diagnosis of IPF in the clinical setting, particularly its differentiation between iNSIP and other interstitial lung diseases (ILDs) is challenging^5,6^. The body of information regarding biomarkers for ILDs is increasing. Some molecules related to the extracellular matrix and dysfunction of epithelial cells and the immune system have been suggested as promising markers for exploring the pathogenesis of IPF and/or its diagnosis, although, in the clinical settings, the use of biomarkers has not been recommended for the diagnosis of IPF^7–9^.

Circulating monocytes mediate essential regulatory and effector functions in innate and adaptive immunity and are divided into three subsets: CD14-strong positive CD16-negative cells (classical monocytes), CD14-strong positive CD16-positive cells (intermediate monocytes), and CD14-weak CD16-positive cells (alternative monocytes)^10^. CD14^strong^CD16^−^ monocytes are the dominant subset and migrate into inflamed tissues to differentiate into tissue macrophages. Recently, circulating monocyte counts have been reported to predict the prognosis of IPF^11,12^. Moreover, the cellular phenotypes of monocytes/macrophages have been associated with ILDs, focusing on the expression of CD163, a hemoglobin scavenging receptor against ROS and an anti-inflammatory marker^13–17^. Very recently, the surface expression of CD163 and S100A9 on classical monocytes has been associated with chronic obstructive pulmonary disease (COPD) and host background factors, such as age and smoking^18^. S100A9 belongs to the S100 family of EF-hand calcium-binding proteins and presents as homodimers and/or heterodimers with S100A8 (S100A8/A9)^19^. S100A9 is a pro-inflammatory marker, and plays roles in the development of inflammation and the production of ROS via the receptor of advanced glycation end products and toll-like receptor-4, although their individual functions are not fully understood^20^. Importantly, S100A9 is expressed on the surface of monocytes as a homodimer, but not as a heterodimer with S100A8 (S100A8/A9), which is predominantly located within the cytoplasm^21^. In the present study, we characterized the expression profiles of S100A9 and CD163 on the cell surface of CD14^strong^CD16^−^ classical monocytes in the quest for diagnostic biomarker for IPF.

## Patients and methods

### Subject selection

Subjects with idiopathic pulmonary fibrosis (IPF) (n = 25) and idiopathic nonspecific interstitial pneumonia (iNSIP) (n = 25) and healthy volunteers (n = 20) were prospectively recruited at Iwate Medical University Hospital from April 2016 until March 2019. Healthy volunteers were consecutively enrolled from among subjects over 50 years of age. IPF and iNSIP who were newly diagnosed in accordance with the criteria outlined by the 2011 consensus statements of the American Thoracic Society, European Respiratory Society, Japanese Respiratory Society, and Latin American Thoracic Association, were consecutively enrolled in our study^22^. Patients receiving corticosteroids, immunosuppressants, and/or anti-fibrotic agents were excluded. Age, sex, smoking status, and pulmonary function tests were assumed as potential confounders in the present study.

### Isolation of peripheral blood mononuclear cells (PBMCs)

Venous blood was collected into vacutainers containing ethylenediaminetetraacetic acid (EDTA). The blood was layered onto FICOLL-PAQUE PREMIUM 1.073 (GE Healthcare, Chicago, IL, USA) and centrifuged to separate PBMCs and plasma. PBMCs were collected, washed with 1% BSA-PBS.

### Flow cytometry analysis

PBMCs were incubated with mixtures of fluorochrome-conjugated antibodies, and identified by flow cytometry. Data acquisition and analysis were performed using a BD CANTO II Flow Cytometer and BD FACS DIVA software (BD Biosciences, Flanklin Lakes, New Jersey). Dead cells were distinguished by 7-amino-actinomycin D (7-AAD, BD Biosciences) staining. Cells were not permeabilized for intra-staining. To identify monocyte lineage cells, the surface markers CD14-PE (Biolegend, San Diego, California, clone HCD14) and CD16-APC/Cy7 (Biolegend, clone 3G8) were used. Pro-inflammatory and anti-inflammatory phenotypes were characterized using S100A9-FITC (BioRad, Hercules, California, clone MAC387) and CD163-APC (Biolegend, clone RM3/1) antibodies (Supplementary Table). S100A9 and CD163 expression levels were determined using a positive dataset for each antibody, identified using a matched concentration of mouse IgG1 kappa isotype control for fluorochrome color (Biolegend, clone MOPC21).

### Pulmonary function tests

Pulmonary function tests were performed within a month prior to flow cytometry analysis. The values of the forced vital capacity (FVC) and forced expiratory volume in 1 s were expressed as percentages of the predicted normal values, calculated according to sex, weight, and age^23^.

### Measurement of KL-6 and SP-D

Krebs von den Lungen-6 (KL-6) concentration was measured with a commercially available chemiluminescent enzyme immunoassay kit (Picolumi KL-6, Eisai, Tokyo, Japan) according to the manufacturer’s instructions. Serum surfactant protein-D (SP-D) concentration was also measured with a commercially available enzyme immunoassay kit (Yamasa-EIA II, Kyowa-Medex, Tokyo, Japan).

### Statistical analysis

The patients’ baseline characteristics are provided as mean ± standard error of the mean. Data are reported for the full cohort. The normality of distribution was estimated using the Kolmogorov–Smirnov test. The statistical significance of differences between the three groups was evaluated by one-way analysis of variance (ANOVA) followed by Steel–Dwass’s post-hoc test. The statistical significance of differences between two groups was evaluated using the Mann–Whitney U test, Fisher's exact test or Chi-square test. Spearman's rank correlation coefficient was used to evaluate the relationships between (1) S100A9^+^ and CD163^+^ circulating classical monocytes and (2) each parameter in a pulmonary function test and SP-D and KL-6. A p value less than 0.05 indicated statistical significance. For each parameter, binary threshold variables to discriminate IPF from iNSIP were analyzed using both unadjusted and adjusted logistic regression analysis, with adjustment variables, including sex, age, smoking status, and FVC (% predicted). Receiver operating characteristic (ROC) curves were plotted for each percentage and differential diagnosis between IPF and iNSIP patients, and the area under the curve (AUC) was calculated. A diagnostic test with an AUC exceeding 0.75 was regarded as contributive^24^. Statistical analyses were performed using the SPSS statistics software (SPSS Inc., Chicago, IL, USA). Adjusted ROC analysis and the comparison of adjusted AUC values between SP-D with and without each parameter were performed via using the statistical software EZR (Easy R) Ver1.52, which is based on R and R commander^25^. The data for pulmonary function tests and serum KL-6/SP-D levels were missing in 6% and 4% of the cohort, respectively. Missing data were removed from data units.

### Ethics approval and consent to participate

The protocol of the present study conformed to the ethical guidelines of the World Medical Association Declaration of Helsinki, and was approved by Iwate Medical University (IRB, H28-12). All subjects provided written informed consent.

## Results

**Figure 1:**
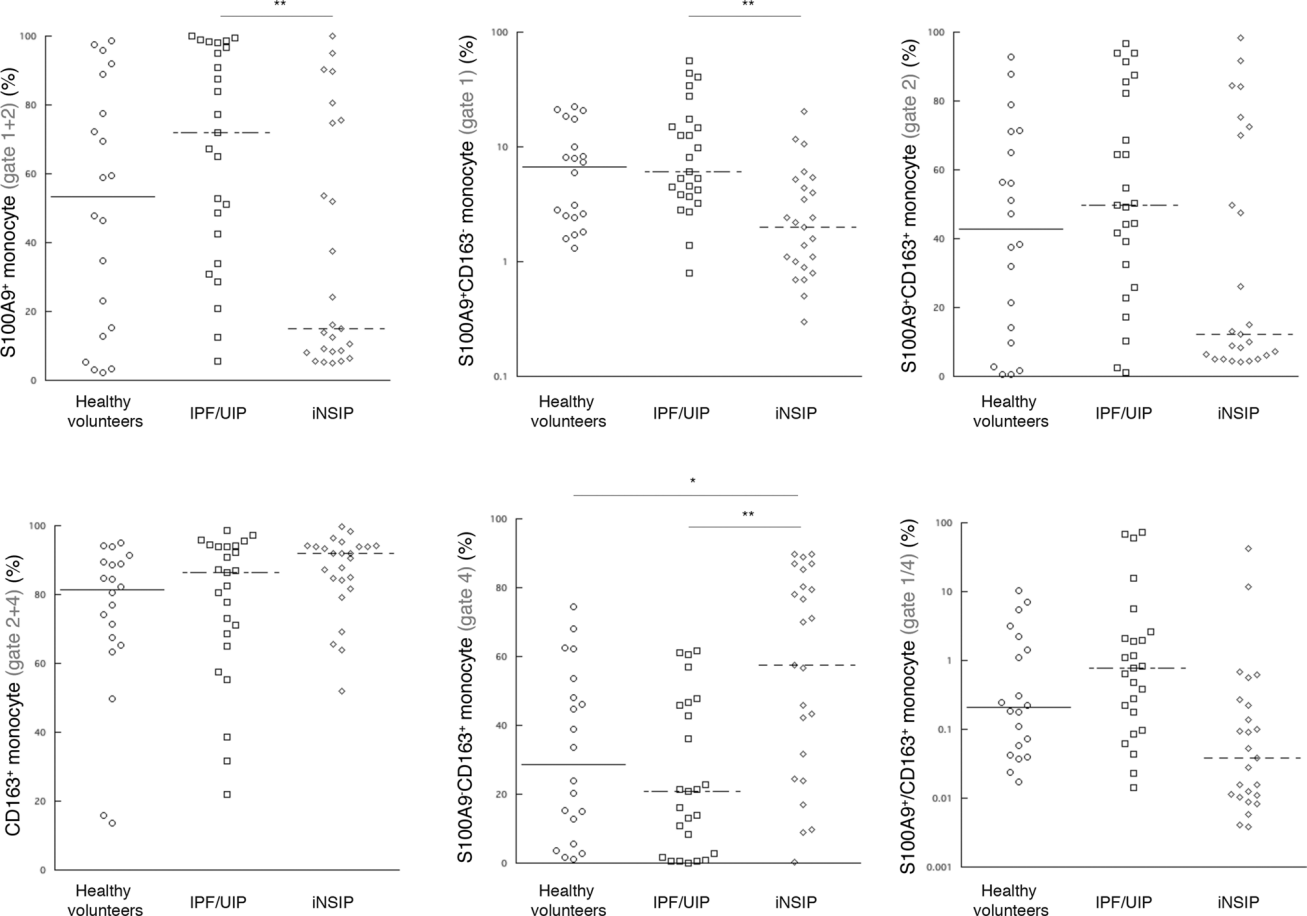
Percentages of S100A9^+^ and CD163^+^ circulating classical monocytes in three clinical groups—patients with idiopathic pulmonary fibrosis, patients with idiopathic nonspecific interstitial pneumonia, and healthy volunteers. (**Upper left**) Percentages of S100A9^+^ monocytes (Gate 1 + 2). (**Upper middle**) Percentages of S100A9^+^CD163^−^ monocytes (Gate 1). (**Upper right**) Percentages of S100A9^+^CD163^+^ monocytes (Gate 2). (**Lower left**) Percentages of CD163^+^ monocytes (Gate 2 + 4). (**Lower middle**). Percentages of S100A9^−^CD163^+^ monocytes (Gate 4). (**Lower right**) S100A9^+^/CD163^+^ monocyte ratio (Gate 1/Gate 4). ∗*p* < 0.05, ∗∗*p* < 0.01, and ∗∗∗*p* < 0.001 by one-way analysis of variance.

**Figure 2:**
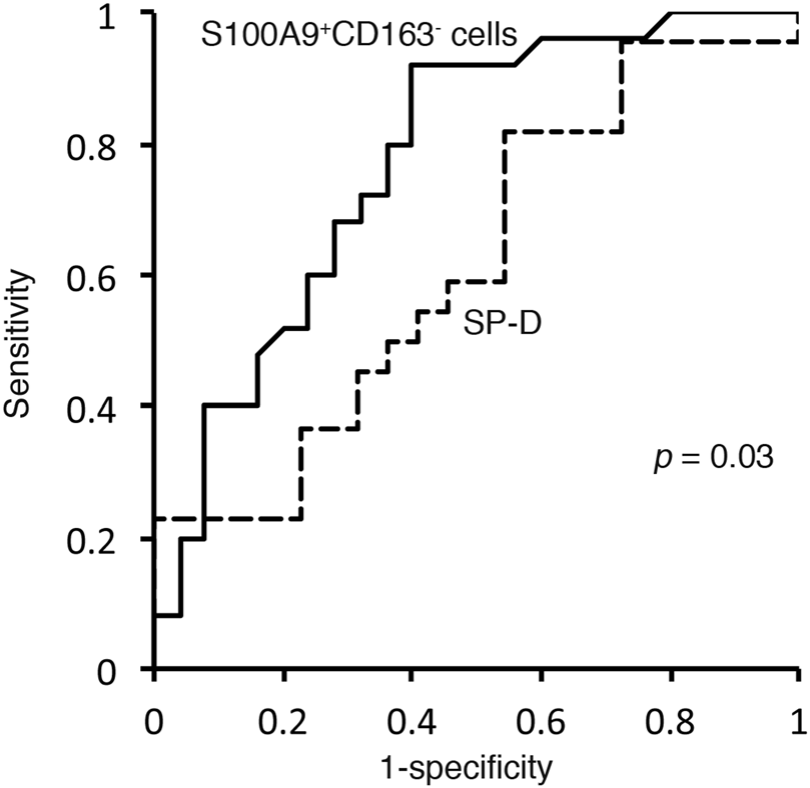
Receiver operating characteristic (ROC) curve analysis to assess the diagnostic value of the percentages of S100A9^+^CD163^−^ cells in circulating classical monocytes for discriminating idiopathic pulmonary fibrosis (IPF) from idiopathic nonspecific interstitial pneumonia (iNSIP). The unadjusted area under the curve (AUC) was 0.802 (95% confidence interval [CI] = 0.687–0.928) for the diagnosis of IPF, which is significantly better than the unadjusted AUC of a current biomarker for IPF, surfactant protein-D (SP-D) (AUC 0.616, 95% CI = 0.446–0.785).

Patient characteristics are shown in Table 1. The smoking status, represented by the pack-year index, showed no difference among the three groups analyzed by one-way ANOVA. We identified three subsets of monocytes, as described above (Supplementary Fig. S1). The ratio of dot plot number of classical monocytes to that of all monocytes did not differ across the three groups (data not shown). We explored the intensity of S100A9 and CD163 expression in classical and non-classical monocytes. Although individuals of delta mean fluorescent intensity (M.F.I) of CD163 and S100A9 showed similar trends between classical and non-classical monocytes, the individual delta M.F.I of S100A9 and CD163 in classical monocytes but not that in non-classical monocytes showed a significant increase in iNSIP as compared to IPF patients (*p* < 0.05 and *p* < 0.05). In addition, the delta M.F.I of CD163 was pronouncedly decreased in non-classical monocytes relative to classical monocytes, while the delta M.F.I of S100A9 was almost equivalent between classical and non-classical monocytes (Supplementary Fig. S2). Furthermore, we analyzed dot plots of S100A9 and CD163 in classical monocytes. Flow cytometry dot plots demonstrated that classical monocytes were divided into four distinct categories: S100A9^+^CD163^−^ (Gate 1), S100A9^+^CD163^+^ (Gate 2), S100A9^−^CD163^−^ (Gate 3), and S100A9^−^CD163^+^ (Gate 4) (Supplementary Fig. S1). The percentages of S100A9^+^ cells (Gate 1 + 2) and S100A9^+^CD163^−^ (Gate 1) showed comparative characteristics among the three groups, and were significantly higher in IPF patients than in iNSIP patients (*p* < 0.01); the percentages of S100A9^+^CD163^−^ (Gate 1) were represented using s log scale (Fig. 1). In contrast, the percentages of S100A9^−^CD163^+^ cells (Gate 4) were significantly increased in iNSIP patients compared to those in IPF patients and healthy volunteers, respectively (*p* < 0.01 and *p* < 0.05, respectively). The percentages of S100A9^+^CD163^+^ cells (Gate 2) and the S100A9^+^CD163^−^ cells (Gate 1)/S100A9^−^CD163^+^ cells (Gate 4) ratio showed no significant differences among the three groups. There was no difference between cellular and fibrotic iNSIP (Supplementary Fig. S3). In non-classical monocytes, the percentages of each gate showed no significant differences among the three groups (data not shown). In IPF patients, the percentages of S100A9^+^CD163^−^ monocytes showed a trend toward a moderate correlation with the serum levels of surfactant protein-D (SP-D) (*r* = 0.4158 [95% CI − 0.02042–0.7191], *p* = 0.051, Table 2). Univariate regression analysis showed that the percentages of S100A9^+^ cells (Gate 1 + 2), S100A9^+^CD163^−^ cells (Gate 1), and S100A9^−^CD163^+^ cells (Gate 4) had statistically significant association with IPF, discriminating it from iNSIP among the four parameters (Table 3). Multivariate logistic regression analysis revealed that the percentage of S100A9^+^CD163^−^ cells (Gate 1) and S100A9^−^CD163^+^ cells (Gate 4) was a diagnostic factor for discriminating IPF from iNSIP, independent from age, sex, smoking status, and %FVC (Table 3). Furthermore, we determined the diagnostic value of each parameter for differentiating IPF from iNSIP. According to the unadjusted ROC-AUC analysis, the percentage of S100A9^+^CD163^−^ cells (Gate 1) showed the best diagnostic value for IPF (ROC-AUC 0.802, 95% CI [0.687–0.928]) and was a significantly better biomarker to discriminate IPF from iNSIP than serum SP-D (0.616, 95% CI [0.446–0.786]) (*p* < 0.05, Fig. 2). There were no significant differences between the adjusted ROC of each parameter of classical monocytes and the serum levels of KL-6 and SP-D.

**Table 1:** Patient characteristics.

	Healthy volunteers (20)	IPF (25)	iNSIP (25)	Healthy volunteers vs. IPF	Healthy volunteers vs. iNSIP	IPF vs. iNSIP
Age	67.0 ± 2.4	69.8 ± 0.3	69.3 ± 2.3	n.s	n.s	n.s
Female (%)	45.0	16.0	48.0	*	n.s	*
Smoker (%)	55.0	68.2	40.0	n.s	n.s	0.08
Pack-years smoking	0.95 ± 0.20	1.44 ± 0.22	0.71 ± 0.20	n.s	n.s	n.s
Current smoker (%)	20.0	26.3	13.0	n.s	n.s	n.s
RF		2	2			n.s
ANA		0	6			**
ARS Ab	–	1	1	–	–	n.s
ScI70/Centromere Ab	–	0	3	–	–	0.065
ANCA		1	0			n.s
FVC	2.94 ± 0.13	2.16 ± 0.12	2.34 ± 0.21	***	***	n.s
%FVC	99.6 ± 4.2	70.2 ± 3.9	79.6 ± 5.0	***	***	n.s
FEV	2.26 ± 0.12	1.85 ± 0.10	1.82 ± 0.16	***	***	n.s
%FEV	91.0 ± 5.4	75.1 ± 3.0	79.3 ± 4.1	***	***	n.s
%DLco	–	73.6 ± 4.4	87.0 ± 6.5	–	–	n.s
LDH	–	266.7 ± 16.7	238.6 ± 12.4	–	–	n.s
KL-6		1179.1 ± 134.5	1133.6 ± 217.5			n.s
SP-D		306.0 ± 46.0	219.5 ± 36.0			0.051

*IPF* idiopathic pulmonary fibrosis, *iNSIP* idiopathic nonspecific interstitial pneumonia, *RF* rheumatoid factor, *ANA* anti-nuclear antibody, *FVC* forced viral capacity, *FEV*_*1.0*_ forced expiratory volume in 1 s, *LDH* lactate dehydrogenase, *KL-6* Krebs von den Lungen-6, *SP-D* surfactant protein-D, – not determined, *n.s* no significance. Data are provided as mean ± standard deviation. *p < 0.05, **p < 0.01, ***p < 0.001.

**Table 2:** Correlation of each parameter of S100A9^+^ and CD163^+^ classical monocytes with serum markers and pulmonary function tests.

	IPF				iNSIP			
	FVC	%FVC	KL-6	SP-D	FVC	%FVC	KL-6	SP-D
S100A9 (%) (Gate 1 + 2)	0.0729	− 0.1508	− 0.3225	0.0457	− 0.1315	0.1548	− 0.0109	− 0.0044
S100A9^+^ CD123^−^ (%) (Gate 1)	0.2877	0.0466	− 0.1859	0.4158	− 0.0240	0.0080	− 0.0584	0.1400
S100A9^−^ CD123^+^ (%) (Gate 4)	0.1074	0.3124	0.0435	− 0.2593	0.2147	0.2059	0.0509	− 0.0436
S100A9^+^ CD123^−^/S100A9^−^ CD123^+^ (Gate1/4)	0.1096	− 0.2185	− 0.2016	0.3281	− 0.1034	− 0.103	0.0073	0.183

*IPF* idiopathic pulmonary fibrosis, *iNSIP* idiopathic nonspecific interstitial pneumonia, *FVC* forced viral capacity, *KL-6* Krebs von den Lungen-6, *SP-D* surfactant protein-D.

**Table 3:** Univariate and multivariate logistic regression analyses and ROC analyses for IPF diagnoses discriminating from iNSIP.

Biomarker	Sens	Spec	Threshold	Unadjusted					Adjusted				
				Exponent	95% CI		p value	AUC	Exponent	95% CI		p value	AUC
KL-6 (U/mL)	77.2	37.5	652	1.0000	0.9993	1.0006	0.94	0.599	1.0230	0.9560	1.0948	0.58	0.713
SP-D (ng/mL)	81.8	45.5	117	0.9965	0.9925	1.0006	0.09	0.616	0.9968	0.9g16	1.0020	0.22	0.629
S100A9 (%)	84.0	52.0	28.6	0.9783	0.9616	0.9952	< 0.05	0.711	0.9812	0.9618	1.0011	0.064	0.765
S100A9^+^CD163^−^ (%)	92.0	60.0	2.7	0.9338	0.8727	0.9991	< 0.05	0.802	0.9151	0.8432	0.9931	< 0.05	0.800
S100A9^−^CD163^+^ (%)	68.0	80.0	24	1.0332	1.0110	1.0559	< 0.01	0.729	1.0345	1.0054	1.0644	< 0.05	0.798
S100A9^+^CD163^−^/S100A9^−^CD163^+^	92.0	52.0	0.044	1.0029	0.9925	1.0134	0.589	0.766	1.0018	0.9872	1.0166	0.815	0.702

*Sens* sensitivity, *Spec* specificity, *CI* confidence interval, *AUC* area under the receiver operating characteristic curve.

## Discussion

In the present study, we found characteristic differences in the expression of the classical monocyte cell surface markers S100A9 and CD163 between patients with IPF and iNSIP. Multiple group comparisons and multivariate logistic regression analysis indicated that IPF and iNSIP show distinct expression profiles of S100A9 and CD163 expression on the surface of classical monocytes. Macrophages in IPF have been immunohistochemically associated with fewer CD163^+^ cells than those in iNSIP, which is consistent with the findings of the present study^24,25^. To our knowledge, this is the first report indicating that the cellular phenotypes of classical monocytes, which form a major population of monocyte lineages, are associated with IPF. These results may encourage further investigations to identify novel clinical biomarkers using monocyte lineages in the context of IPF and provide new insight into the pathogenic roles of circulating monocytes in both IPF and iNSIP.

A gradually increasing body of literature is becoming available regarding the association between interstitial lung diseases and monocyte phenotypes. Trombetta et al. reported that increased rates of circulating pro-inflammatory M1/anti-inflammatory M2 mixed CD14^+^ monocytes were more associated with systemic sclerosis with interstitial lung diseases than with systemic sclerosis without interstitial lung diseases^13^. Consistently, in the present study, different trends were observed between the percentages of CD163^+^S100A9^+^ and CD163^+^S100A9^−^ cells (Gate 2 + 4) and those of CD163^+^S100A9^−^ cells (Gate 4). Taken together, these findings highlight the importance of the simultaneous determination of pro-inflammatory and anti-inflammatory phenotypes in the estimation of circulating monocytes. Greiffo et al. showed that circulating non-classical monocytes were significantly decreased in ILDs, including nonspecific interstitial pneumonia, hypersensitivity pneumonitis, and collagen vascular disease-associated interstitial pneumonia compared with those in healthy volunteers^14^. In contrast, non-classical monocyte counts and the expression of CX3CR1 in monocytes were higher in lung. In addition, CD163 expression on non-classical monocytes showed a significant increase in ILDs patients than in healthy volunteers. In the present study, CD163 expression on non-classical monocytes did not show significant differences among the three groups. In contrast, the percentages of S100A9^−^CD163^+^ cells on classical monocytes were significantly increased in iNSIP patients than in both healthy volunteers and IPF. Of note, the levels of delta M.F.I with respect to the expression of CD163 on classical monocytes appeared to be higher than those on non-classical monocytes, consistent with the results reported by Greiffo and coworkers. These findings suggested the importance of CD163 signals on classical monocytes rather than non-classical monocytes.

Very recently, the expression of S100A9 and CD163 on circulating monocytes has been associated with age, smoking, and COPD^18^. In the present study, multivariate logistic regression analysis revealed that the percentage of S100A9^+^CD163^−^ cells (Gate 1) and S100A9^−^CD163^+^ cells (Gate 4) was a diagnostic factor for discriminating IPF from iNSIP, independently from the smoking status, in addition to age, sex, and %FVC. Moreover, the percentage of S100A9^+^CD163^−^ cells (gate 1) in IPF tended to correlate moderately with the SP-D serum levels. In contrast, no parameters were correlated with disease activity and/or severity of iNSIP. Taken together, these results imply that circulating classical monocytes play multiple pathogenic roles in heterogeneous fibrotic conditions.

ROS have been reported to contribute to tissue damage in IPF, and S100A9 homodimers play pro-inflammatory roles and the production of ROS, while CD163 receptors on macrophages scavenge hemoglobin via heme-oxygaenase-1 pathways, and inhibit the production of ROS^17^. We dare to speculate that S100A9-dominant classical monocytes might perpetuate tissue damage from lung injury in IPF as precursors of S100A9^+^ macrophages on alveolar spaces, while CD163-dominant monocytes rather play a protective role with reversibility against lung injury in iNSIP, but not profibrosis that is classically characterized as a major role of M2 polarized macrophages. However, we cannot exclude the possibility that cellular phenotypes of classical monocytes are an epiphenomenon caused by the underlying pathologies.

The number of potential diagnostic biomarkers for IPF is growing: for instance, the serum levels of matrix metalloproteinase (MMP)-7, MMP-28, SP-D, and S100A9 have been explored as IPF biomarkers^26–28^. The serum levels of S100A9 and MMP28 are promising biomarkers for discriminating between IPF and other ILDs. In particular, Hara et al. reported a ROC-AUC value of 0.92 for serum S100A9 in the discrimination IPF from iNSIP^29^. In contrast, Bennett et al. reported no difference in the serum levels of S100A9 homo-dimer levels between IPF and fibrosing iNSIP patients, which is inconsistent with our results^27^. However, while S100A9 homo-dimers are rigid in the plasma membrane, they are unstable in the serum, which might explain the discrepancy between our results and those of Bennett et al.^30^. In the present study, multivariate logistic regression analysis suggested that the increased percentages of S100A9^+^CD163^−^ cells (Gate 1) and S100A9^−^CD163^+^ cells (Gate 4) contributed significantly (as per the parameters in the adjusted ROC-AUC analysis) towards the discrimination between IPF and iNSIP. In addition, the unadjusted ROC values of S100A9^+^CD163^−^ cells (Gate 1) were significantly higher than those of serum SP-D. Our study therefore indicates that S100A9 and CD163 in classical monocytes could be potentially useful diagnostic markers for IPF.

This study has some limitations. First, the sample size is relatively small. Additionally, missing data were removed. Thus, further studies on a larger cohort of patients are needed to validate these preliminary results. Second, we could not determine the properties of S100A9^+^CD163^+^ cells (Gate 2). When the three groups were compared, the results for Gate 2 were similar to those of Gate 1 but not to those of Gate 4. A similar trend was also found in the previous study regarding COPD^18^. We speculate that the properties of S100A9^+^CD163^+^ cells are entirely different from those of S100A9^−^CD163^+^ cells. This should be validated by the comparison of gene expression profiles. Third, we used only S100A9 and CD163 in circulating monocytes as pro-inflammatory and ant-inflammatory biomarkers, respectively, and found pro-inflammatory phenotypes in classical monocytes of IPF patients, and anti-inflammatory phenotypes in those of iNSIP patients. However, it remains to be elucidated whether other pro-inflammatory and anti-inflammatory surface markers can confirm these phenotypes. Forth, some papers have recently reported that circulating monocyte counts could predict the prognoses of IPF. However, we did not determine the total circulating monocyte counts in the present study; we only focused on the percentage of S100A9^+^ and CD163^+^ cells. Therefore, further studies are needed to validate our data within circulating classical monocytes.

## Conclusion

In conclusion, we used a clinical cohort to identify differences in the cell-surface levels of S100A9 and CD163 in classical monocytes by flow cytometry. We found monocyte phenotypes that were specific to patients with IPF and those with iNSIP that had potentially better discriminatory ability for these clinical groups than other existing biomarkers for IPF. An effective diagnostic biomarker for the early detection of IPF would allow for timeous medical interventions that could improve patient outcomes for this important disease.

## Supplementary Information

Supplementary Information 1.

Supplementary Figure 1.

Supplementary Figure 2.

Supplementary Figure 3.

## Data Availability

All of the de-identified individual participant data collected during the study are available. Proposal should be directed to yamam@iwate-med.ac.jp.

## Abbreviations

IPF: Idiopathic pulmonary fibrosis
iNSIP: Idiopathic nonspecific interstitial pneumonia
ILDs: Interstitial lung diseases
COPD: Chronic obstructive pulmonary disease
PBMCs: Peripheral blood mononuclear cells
FVC: Forced vital capacity
KL-6: Krebs von den Lungen-6
SP-D: Surfactant protein-D
ANOVA: One-way analysis of variance
ROC: Receiver operating characteristic
AUC: Area under the curve
MMP: Matrix metalloproteinase
