# Supplementary material for: S100A9/CD163 expression profiles in classical monocytes as biomarkers to discriminate idiopathic pulmonary fibrosis from idiopathic nonspecific interstitial pneumonia
Source: Sci Rep. 2021 Jun 9;11:12135. doi: 10.1038/s41598-021-91407-9 (PMC8190107; doi:10.1038/s41598-021-91407-9)
Supplement: Supplementary file 1 — Supplementary Information 1. [file 41598_2021_91407_MOESM1_ESM.docx]

**S100A9/CD163 expression profiles in classical monocytes** **as biomarkers to discriminate idiopathic pulmonary fibrosis from idiopathic nonspecific interstitial pneumonia**

Masahiro Yamashita^1*^, Yuh Utsumi^1^, Hiromi Nagashima^1^, Hiroo Nitanai^1,2^, and Kohei Yamauchi^1,3^

^1^Department of Pulmonary Medicine, Allergy and Immunological Diseases, Iwate Medical University School of Medicine, Shiwa, Japan

^2^Internal Medicine, Ishidoriya Medical Center, Hanamaki, Japan

^3^Internal Medicine, Takisawa Chuo Hospital, Takisawa, Japan

*Address for correspondence and reprint requests:

Masahiro Yamashita, MD

Department of Pulmonary Medicine, Allergy and Immunological Diseases, Iwate Medical University, 1-1-1 Idaidouri, Yahabacho, Shiwa, 028-3694, Japan

Telephone: +81-19-651-5111

Fax: +81-19-907-6674

E-mail: [yamam@iwate-med.ac.jp](mailto:yamam@iwate-med.ac.jp)

**Supplementary figure legends**

**Supplementary figure 1.** Representative flow cytometric analyses for peripheral blood circulating monocytes. Representative plots illustrate the gating strategy in samples obtained from healthy volunteers. Cells obtained from the peripheral blood monocytes (PBMCs) were used for analyses, and dead cells, doublets and debris were excluded as represented in gating strategy (Top). CD14^+^CD16^-^—classical monocytes, CD14^+^CD16^+^—intermediate monocytes, and CD14^diminish^CD16^+^—alternative monocytes were identified (Second Left). Dot plots represent primary antibodies against S100A9 and CD163 in classical monocytes. (Second Right) Healthy volunteer. (Third) Idiopathic pulmonary fibrosis (IPF) patient. (Bottom) Idiopathic nonspecific interstitial pneumonia (iNSIP) patient. FSC: Forward-scattered light, SSC: Side-scattered light, A: area, H: height, W: Width, 7ADD: 7-amino-actinomycin D.

**Supplementary figure 2.** The results of delta mean fluorescent intensity (M.F.I). (A) Delta M.F.I of S100A9 expression on classical monocytes. (B) Delta M.F.I of CD163 expression on classical monocytes. (C) Delta M.F.I of S100A9 expression on non-classical monocytes. (D) Delta M.F.I of CD163 expression on non-classical monocytes. Data are presented as the mean ± standard error of the mean. ^∗^*p* < 0.05 by one-way analysis of variance.

**Supplementary figure 3**. Rates of S100A9^+^ and CD163^+^ circulating classical monocytes between cellular and fibrotic iNSIP. (A). Rates of S100A9^+^ monocytes (Gate 1+2). (B). Rates of S100A9^+^CD163^-^ monocytes (Gate 1). (C). Rates of S100A9^+^CD163^+^ monocytes (Gate 2). (D). Rates of CD163^+^ monocytes (Gate 2+4). (E). Rates of S100A9^-^CD163^+^ monocytes (Gate 4). (F). Ratios of S100A9^+^/CD163^+^ monocytes (Gate 1/Gate 4). Data are presented as the mean ± standard error of the mean values, and analyzed by the Mann–Whitney U test.

**Supplementary table**. Primary antibodies used
