# Supplementary figures and images for: S100A9/CD163 expression profiles in classical monocytes as biomarkers to discriminate idiopathic pulmonary fibrosis from idiopathic nonspecific interstitial pneumonia
Source: Sci Rep. 2021 Jun 9;11:12135. doi: 10.1038/s41598-021-91407-9 (PMC8190107; doi:10.1038/s41598-021-91407-9)

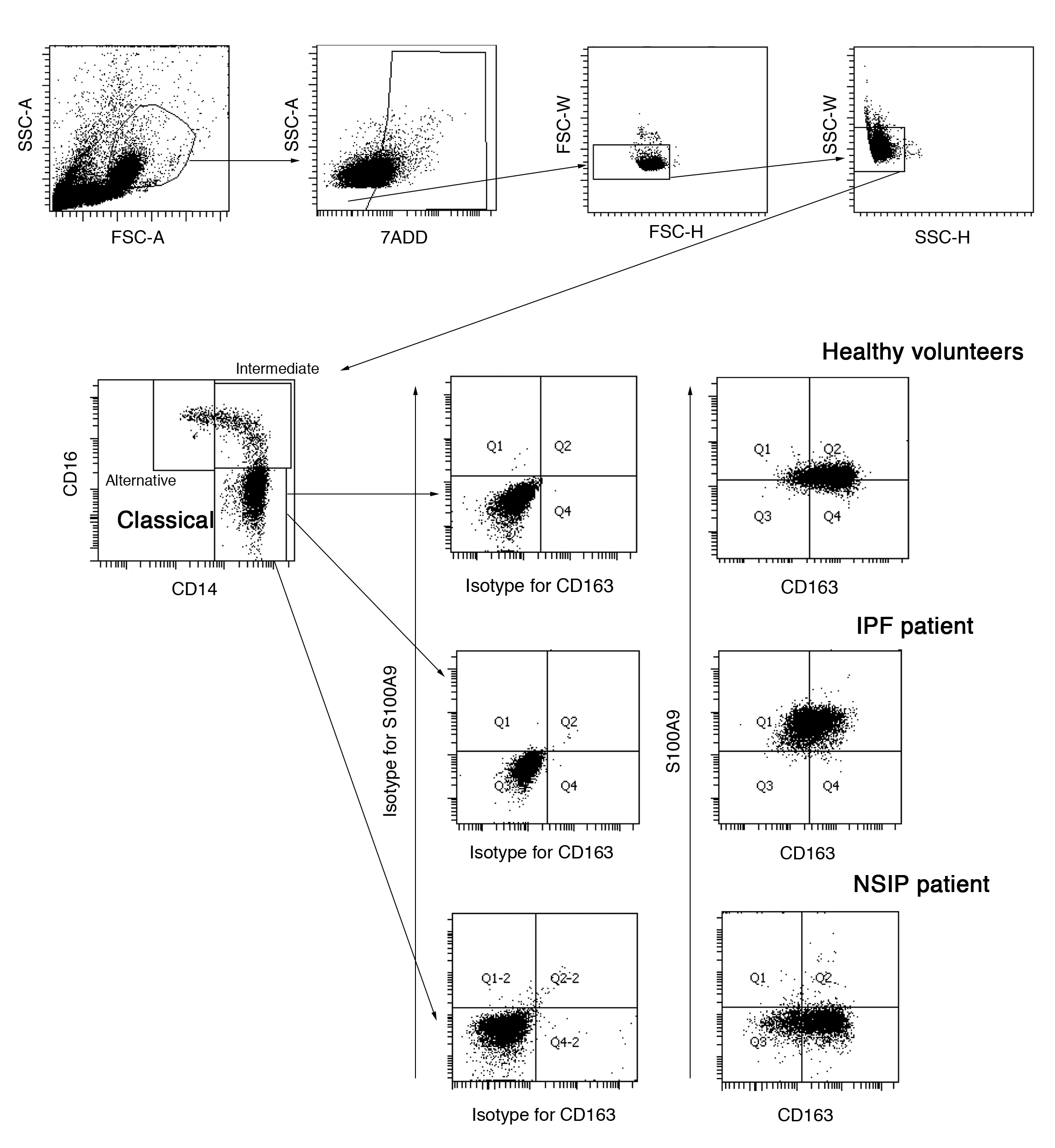

Supplement: Supplementary file 2 — Supplementary Figure 1. [file 41598_2021_91407_MOESM2_ESM.tif]

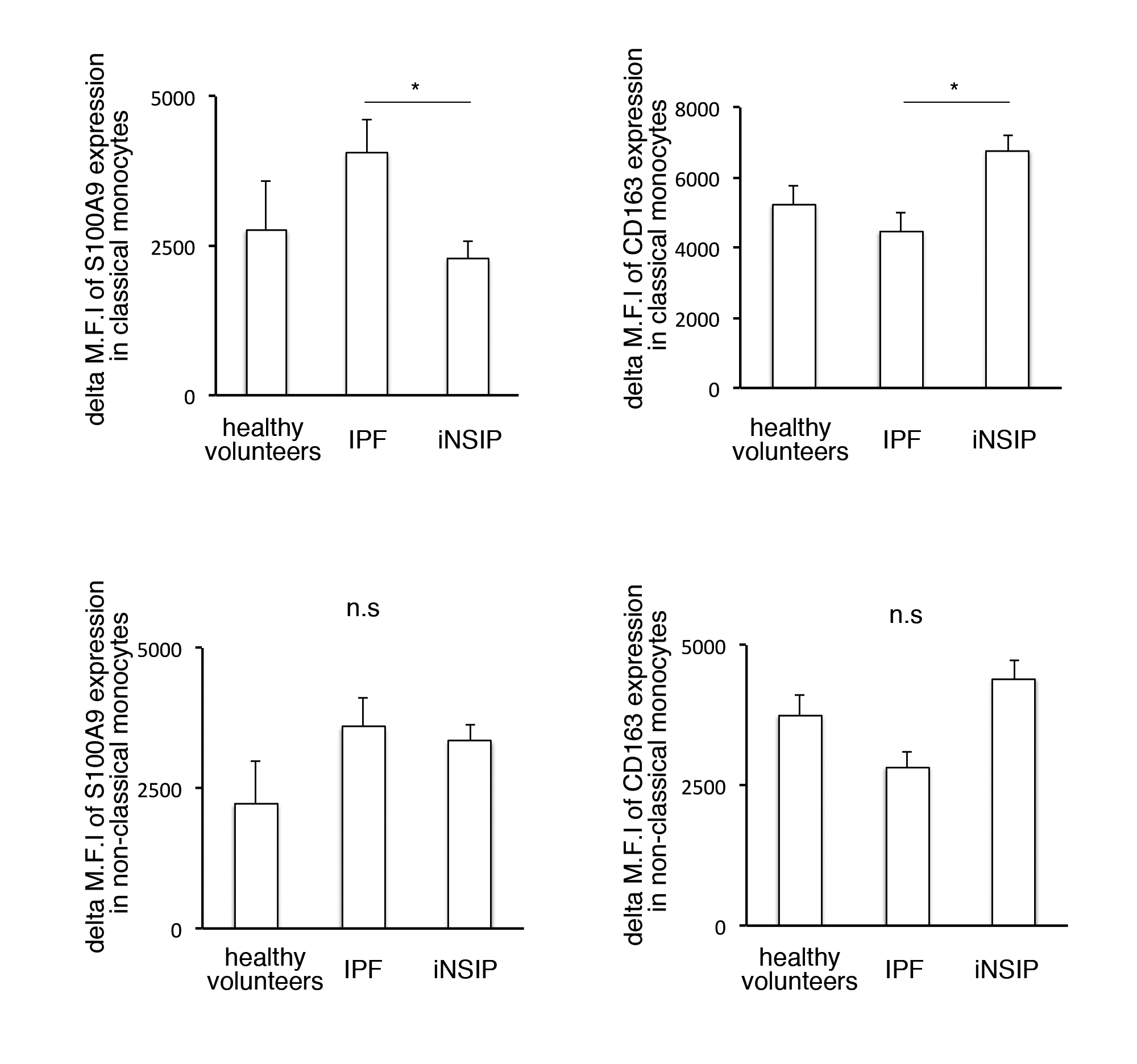

Supplement: Supplementary file 3 — Supplementary Figure 2. [file 41598_2021_91407_MOESM3_ESM.tif]

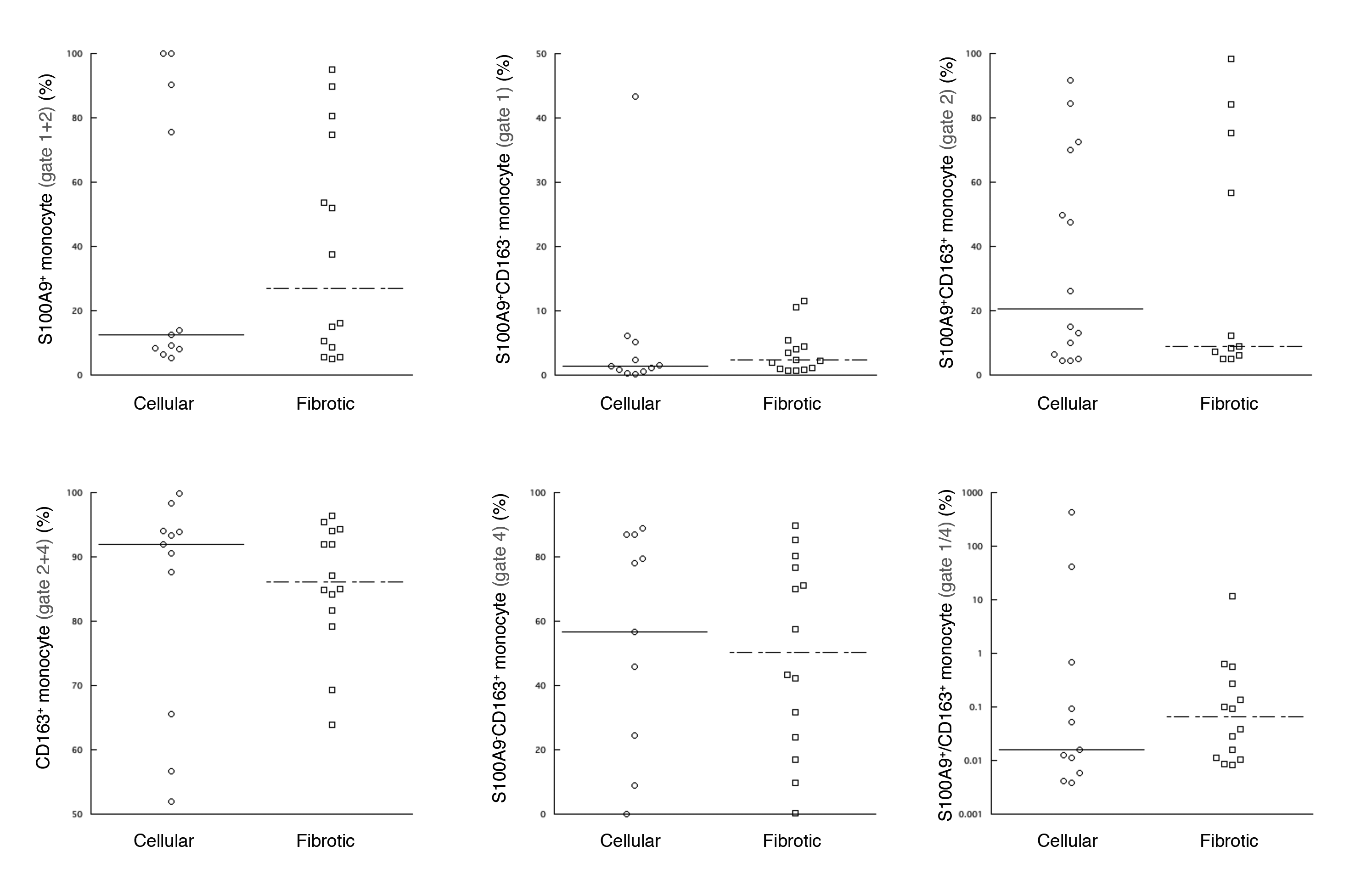

Supplement: Supplementary file 4 — Supplementary Figure 3. [file 41598_2021_91407_MOESM4_ESM.tif]
